# Post-Operative Pain After Knee Arthroscopy and Related Factors

**DOI:** 10.2174/1874325000802010110

**Published:** 2008-06-13

**Authors:** G.I Drosos, N.I Stavropoulos, A Katsis, K Kesidis, K Kazakos, D.-A Verettas

**Affiliations:** 1 Department of Orthopaedic Surgery, Medical School, Democritus University of Thrace, University General Hospital of Alexandroupolis, 68100 Alexandroupolis, Greece; 2 Department of Orthopaedic Surgery, General Hospital of Kalamata, Kalamata, Greece; 3 School of Social Science, University of Peloponnese, Korinthos, Greece; 4 Department of Anesthesiology, Attikon Hospital, Athens, Greece

## Abstract

The aim of this study was to explore the intensity of post-arthroscopy knee pain during the first 24 hours, and to study the influence of pre-operative pain, tourniquet time and amount of surgical trauma on post-arthroscopy pain. In 78 male patients that underwent elective arthroscopic menisectomy or diagnostic arthroscopy of the knee, preoperative and post-operative pain were registered using the Visual Analogue Scale. Variance for repeated measures and for independent observations was analysed. Supplementary analgesia was required for 23% of the patients, more often in the recovery room and between 2 and 8 hours postoperatively. Of all factors analyzed, only time was statistically significant in determining the level of post-operative pain. Supplementary analgesia was required only in patients that underwent operative arthroscopy, and more often in patients with tourniquet time of more than 40 minutes. In conclusions, post-operative time is the most significant factor related to the post-arthroscopy knee pain.

## INTRODUCTION

Knee arthroscopy is a very common procedure and very often is performed as day-case surgery. It seems that ambulatory arthroscopic surgery of the knee is preferred by the majority of properly selected and well informed patients [1]. It has been reported that a significant number of patients have moderate to severe pain 24 hours after ambulatory surgery in general and knee arthroscopy in particular [2,  3], and pain affects the patient’s activity level and satisfaction [3].

In an effort to provide an effective, safe and long lasting post-arthroscopy analgesia, several studies using different drugs and regimes have been published during the last two decades. Intra-articular administration of local anaesthetics has been widely used but some studies have questioned their efficacy [4-11]. The same applies for intra-articular morphine [4,  12-18], for a combination of a local anaesthetic and morphine [19,  20], as well as for pre-emptive analgesia [11,  21].

Several clinical trials reported the effects on post-operative analgesia of various drugs or regimes and numerous factors have been implicated to influence post-arthroscopy pain: anaesthetic technique, residual effects of peri-operative analgesia, sensitivity of the methods for postoperative pain registration, pre-operative pain level, the amount of surgical trauma (i.e. diagnostic arthroscopy or arthroscopic surgery), the use and duration of tourniquet ex-sanguination, the experience of the surgeons, the sex of the patient and the post-operative activity level of the patients [4,  8,  12,  17,  20,  22-26].

The aim of this prospective study was to explore the true incidence and intensity of post-arthroscopy pain and the influencing factors without a specific regime for post-arthroscopy analgesia.

## PATIENTS AND METHODS

In an effort to reduce confounding factors it was decided to include only male patients, treated by one surgeon, using one anaesthetic technique, the same post-operative activity level of the patients and no pre-emptive or intra-operative analgesia apart from Remifentanil. Therefore the variables in this study were the pre-operative pain level, the amount of surgical trauma and the tourniquet time.

Male patients scheduled for elective knee arthroscopy, with normal knee radiographs, ASA I, that underwent diagnostic arthroscopy or arthroscopic meniscectomy participated in this study. The exclusion criteria were (a) previous knee surgery (b) chronic knee pain, or history of arthritis (c) daily intake of steroids (d) recent intake of non-steroidal anti-inflammatory drugs or opioids.

All patients were instructed preoperatively in the use of the 10-cm Visual Analogue Scale (VAS) for pain, 0=no pain to10=the worst pain [27]. Pre-operative pain was assessed using the VAS with the patient performing five active tests: (a) straight leg raising, (b) knee flexion with the patient lying supine, (c) knee extension with the patient sitting on the couch, (d) knee flexion with the patient sitting on the couch, and (e) five steps walking. The average score of these five tests was used as the pre-operative pain score.

All operations were performed by the same surgeon (GID), under general anaesthesia using the same anaesthetic protocol. No pre-medication was used. General anesthesia was induced with Propofol 2 mg/kg, Remifentanil 0.1 μg/kg and Vecuronium 0.1 mg/kg, and maintained with Propofol 0.06 mg/kg/h and Remifentanil infusion as needed, with the patients breathing N2O 70% and O2 30% through endotracheal intubation. A pneumatic tourniquet was inflated at a pressure of 300 mm Hg in all patients, after leg elevation for 3 minutes.

The patients were evaluated by VAS at 2,4,6,8,12,16,24 hours after surgery. In the recovery room patients received morphine 20 mg intravenously when the VAS exceeded 3 and this was registered. Postoperatively the patients commenced isometric quadriceps exercises as soon as they returned to the ward and mobilised on crutches when they felt comfortable. Supplementary analgesia - pethidine 0.5 mg/kg intramuscularly- was given at patients request and the time of administration was registered. All the patients stayed in the hospital for at least 24 hrs before discharge.

## STATISTICAL ANALYSIS

Pain scores at 2, 4, 6, 8, 12, 16, 20 and 24 hours were analyzed in relation to type of arthroscopy (diagnostic or therapeutic), pre-operative pain level (POPL): Low (average VAS: < 3) / Middle (average VAS: 3-6) / High (average VAS: > 6), tourniquet time: Low (< 40 minutes) / High ( > 40 minutes) and supplementary analgesia.

The statistical methodology included analysis of variance for repeated measures and for independent observations [28,  29]. Statistical significance was established for a P-value of less than 0.05.

## RESULTS

Seventy-eight male patients were included in the study. Sixty-five underwent arthroscopic meniscectomy and 13 had a diagnostic procedure (Table **1**).

The average pain was higher during the first 2 hours, followed by a plateau until the 8th hour, decreased until the 16th hour and then reached again a plateau up to the 24th hour (Fig. **1A**).

Eighteen patients (23.1%) required supplementary analgesia, and seven of them received this analgesia twice. The supplementary analgesia was required more often in the recovery room, followed by the 8th and the 2nd postoperative hour (Fig. **1B**). It is worth noting that all patients were mobilized between the 6th and 8th hours post-operatively.

Patients with middle pre-operative pain level (POPL) exhibit on average more post-operative pain than patients in the low POPL, and this difference was significant during the first 6 post-operative hours, while the ones belonging to the high POPL have a mixed post-operative pain behaviour (Table **1**, Fig. **2A**). Supplementary analgesia was required more often in patients with middle POPL (Fig. **2B**).

Diagnostic arthroscopy caused on average less post-operative pain than arthroscopic meniscectomy, but the difference was not significant (P-value >0.05), (Fig. **3A**). Supplementary analgesia was required only for the patients who underwent arthroscopic meniscectomy (Fig. **3B**).

Patients with low tourniquet time exhibit on average less post-operative pain compared to patients with high tourniquet time but again the difference was not significant (P-value >0.05), (Fig. **4A**). Supplementary analgesia was required more often in patients with long tourniquet time than in patients with short tourniquet time (Fig. **4B**).

Not surprisingly, patients that were administered supplementary analgesia postoperatively exhibit on average more pain than those that managed without, and this different pain susceptibility remains wide throughout the 24-hour period.

### Differences Over Time (Repeated Measures Analysis)

The effect of time elapsed since the procedure is statistically significant in determining the level of post-operative pain (F=14, df =4.36, P<0.001). More specifically, the average pain demonstrates a downward trend as time passes (Fig. **1A**).

The interactions of time with the following variables were not significant: (a) pre-operative pain level (F=1.80, df=8.73, P=0.07), (b) the type of procedure (F=1.92, df=4.37, P=0.10), and (c) tourniquet time (F=1.27, df=4.36, P=0.28).

Furthermore, the interaction of time with supplementary analgesia and the three-way interaction among time, POPL and tourniquet time is statistically significant (F=2.98, df=4.37, P=0.017 and F=3.07, df=8.73, P=0.002 respectively).

Regarding the three-way interaction, we observe that within each POPL, the post-operative pain level is almost always higher when the tourniquet time is high. However, this difference varies according to time. The worsening effect of a prolonged tourniquet time on post-operative pain is experienced in the early period (up to the 8th hour) by patients with a low POPL, later (after the 16th hour) by those with middle POPL and between the 8th and the 24th hour by those with a high POPL.

### The Average Post-Operative Pain (Analysis of Variance)

The mean post-operative pain is significantly affected by the fact that supplementary analgesia was given to some patients (P-value < 0.001). More specifically, it is estimated that patients that were administered supplementary pethidine analgesia had a higher post-operative pain by 1.39 units provided that the other factors (type of procedure, tourniquet time, pre-operative pain level) remain the same.

## DISCUSSION

The primary aim of this study was to explore the true incidence and intensity of post-arthroscopy pain for 24 hours. The fact that the patients stayed in the hospital during the entire period of the study has the benefit of a more accurate registration of the pain scores and the required supplementary analgesia, and allows a better control of a similar postoperative level of patient activity [12].

Our patients were not just instructed preoperatively in the use of the 10-cm VAS for pain as in most studies, but actually learned how to use this scale as they assessed their pre-operative pain. It has been suggested that patients who learn to assess their pain and communicate their analgesic needs will have more control over the dose and delivery of analgesic agents regardless of the route of administration [30].

The anaesthetic technique and analgesics or NSAIDs used peri-operatively in particular, may affect the post-operative pain by residual analgesic effect [4,  17,  26]. In order to minimize this residual analgesic effect, apart from Remifentanil, no other analgesia or NSAIDs were used peri-operatively in this study. Remifentanil provides effective analgesia and sedation, with a rapid onset and a short duration of action due to its rapid hydrolysis by blood and tissue esterases [31,  32]. The context-sensitive half-life remains very short (3 to 4 minutes), independent of the duration of infusion [31,  32]. Thus the residual analgesic effect, if any, would be minimal.

The influence of some factors that may affect the post-arthroscopy pain, such as the experience of the surgeons, the sex of the patient, and the post-operative activity level of the patients, was the same in this study. All operations were performed by the same surgeon, only male patients were included in the study, and by having the patients in the hospital the entire study period the patient’s postoperative level activity was similar.

### Post-Arthroscopy Pain Levels

Only 23.1% of the patients in this study required supplementary analgesia. This is in agreement with recent studies where it was found that a significant proportion of patients have only very mild or mild after knee arthroscopic procedures [24,  33-35].

The post-arthroscopy pain intensity may be an important factor when the analgesic effect of various drugs or regimes is studied in clinical trials. The post-arthroscopy pain intensity may influence the sensitivity of methods for postoperative pain registration [24]. It has been suggested that that lower pain intensity might be responsible for low study sensitivity due to weak pain stimulus, since postoperative analgesic affects of intra-articular morphine were found only in a subgroup of patients with higher pain intensity in the immediate post-anaesthetic period [24]. Therefore post-arthroscopy pain intensity may be a confounding factor, reducing assay sensitivity when all patients are included [33].

The results of this study showed that the effect of time is the only statistically significant factor in determining the level of post-operative pain.

The post-arthroscopy pain was found to be more pronounced during the first 8 post-operative hours. These findings should be expected since postoperative pain is at its peak immediately after surgery and becomes less severe with time [30].

Although the need for supplementary analgesia was greater in the recovery room, and during the first 8 post-operative hours, our patients continued to experience some pain during the rest of the 24 hours period, indicating the need for analgesia for at least 24 hours.

### Pre-Operative Pain

Some authors found that the pre-operative level of discomfort was the most significant predictor or determinant of post-operative discomfort [8,  17]. Also low post-operative pain scores were found in patients with little pre-operative pain and small surgical trauma [4]. Others found that postoperative pain was not related to the pre-operative pain scores [20].

In this study it was found that the post-operative pain was on average higher in patients with a middle pre-operative pain level (POPL) than in patients with a low POPL, and this difference was significant during the first 6 post-operative hours, while the ones belonging to the high POPL have a mixed post-operative pain behaviour. Nevertheless supplementary analgesia was required more often in patients in the middle POPL than in the other two groups.

### Amount of Surgical Trauma

Joshi *et al*. (1992) [14] stated that the post-arthroscopy pain seems to be unrelated to any intra-articular procedure which may be carried out and the results of some studies agree with that [12,  8,  20]. In another study, low post-operative pain scores were found in patients with little pre-operative pain and small surgical trauma [4].

Although the difference was not statistical, patients who underwent arthroscopic meniscectomy exhibit on average more post-operative pain than those in the diagnostic arthroscopy group, and supplementary analgesia was required only in patients that underwent arthroscopic meniscectomy.

### Tourniquet Time

Previous studies have found that post-arthroscopy pain is not related to the use of a tourniquet [8,  22] or the duration of tourniquet (tourniquet time) [8,  20]. In another study an increase in post-arthroscopy pain was found in those patients where the tourniquet time was more than 30 minutes [23]. In our study it was found that patients with a tourniquet time more than 40 minutes experienced on average more post-operative pain, and required more often supplementary analgesia compared to patients with tourniquet time less than 40 minutes.

In conclusion, according to the results of this study, the only significant factor related to the level of post-arthroscopy pain was the postoperative time elapsed since surgery. Statistical analysis of the post-operative scores did not show significant difference for the other factors, but supplementary analgesia was required (a) only in patients that underwent arthroscopic meniscectomy, (b) mostly when the tourniquet time exceeded 40 minutes and (c) the preoperative pain level was in the middle range.

## Figures and Tables

**Fig. (1A):**
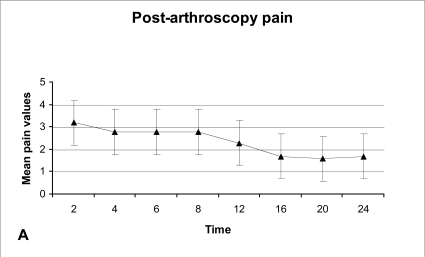
Post-arthroscopy pain during the first 24 hours.

**Fig. (1B):**
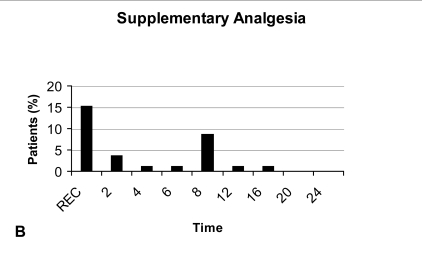
Supplementary analgesia during the first 24 hours. R: Recovery room.

**Fig. (2A):**
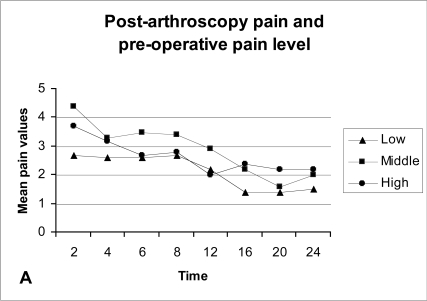
Average post-arthroscopy pain and pre-operative pain level (POPL). Low: POPL < 3. Middle: POPL 3-6. High: POPL > 6.

**Fig. (2B):**
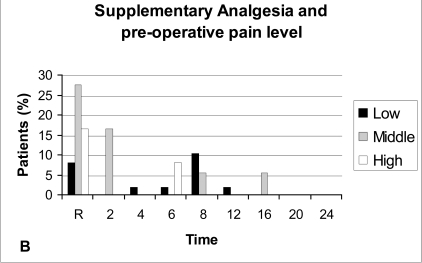
Supplementary analgesia and pre-operative pain level (POPL). Low: POPL < 3. Middle: POPL 3-6. High: POPL > 6.

**Fig. (3A):**
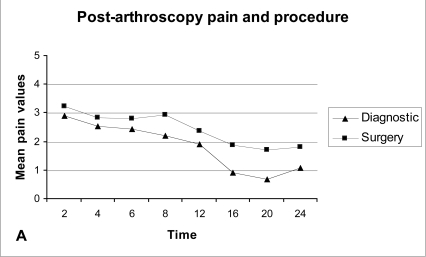
Average post-arthroscopy pain and type of procedure.

**Fig. (3B):**
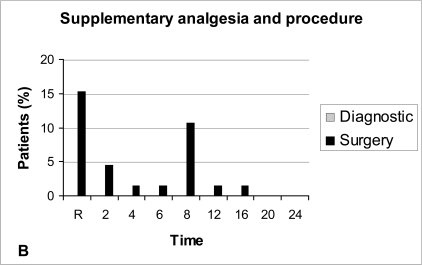
Supplementary analgesia and type of procedure.

**Fig. (4A):**
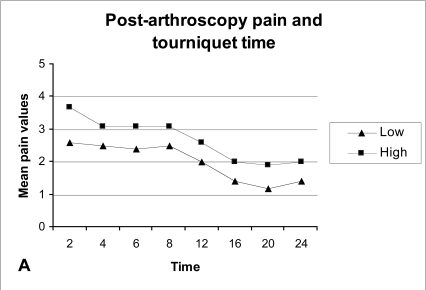
Average post-arthroscopy pain and tourniquet time. Low: ≤40 minutes. High: >40 minutes.

**Fig. (4B):**
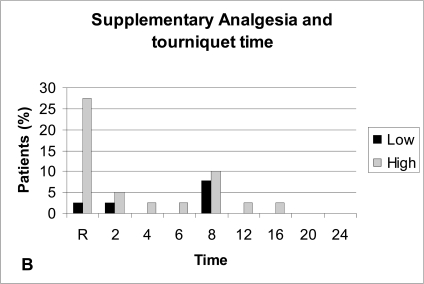
Supplementary analgesia and tourniquet time. Low: ≤40 minutes. High: >40 minutes.

**Table 1.** Demographic and Clinical Data of 78 Male Patients

Demographic Data	
Age: Years (SD)	25.4 (6.11)
Knee: R/L	45/33
**Clinical Data**
Type of procedure: Diagnostic/Surgery	13/65
Pre-operative pain level (POPL): Low (≤ 3) / Middle (3-6) / High (≤ 6)	48/18/12
Tourniquet time categories: Low (≤ 40 minutes) / High (> 40 minutes)	38/40
Supplementary analgesia (given/not given)	18/60
